# Association between dietary antioxidant capacity and type 2 diabetes mellitus in Chinese adults: a population-based cross-sectional study

**DOI:** 10.1186/s12986-024-00786-z

**Published:** 2024-03-29

**Authors:** Xiaoxia Li, Yixuan Xue, Yadi Zhang, Qingan Wang, Jiangwei Qiu, Jiaxing Zhang, Chan Yang, Yi Zhao, Yuhong Zhang

**Affiliations:** 1 Key Laboratory of Environmental Factors and Chronic Disease Control, Ningxia Medical University, 750004 Yinchuan, China; 2 NHC Key Laboratory of Metabolic Cardiovascular Diseases Research, Ningxia Medical University, 750004 Yinchuan, China; 3 School of Public Health of Ningxia Medical University, 750004 Yinchuan, China

**Keywords:** Type 2 diabetes mellitus, Dietary total antioxidant capacity, Dietary antioxidant quality scores

## Abstract

**Background:**

Higher intakes of dietary antioxidants have been linked to a lower type 2 diabetes mellitus (T2DM) risk. However, few studies have comprehensively examined the overall dietary antioxidant capacity, assessed by dietary antioxidant quality scores (DAQS) and dietary total antioxidant capacity (DTAC), related to T2DM risk, especially in populations consuming relatively monotonous diets. This study aimed to evaluate the associations of DAQS, DTAC, and T2DM among rural Chinese adults.

**Methods:**

Data from 12,467 participants from the Natural Population Cohort of Northwest China: Ningxia Project was analyzed. Dietary intake was assessed using a validated semi-quantitative food frequency questionnaire. DAQS were calculated based on vitamins A, C, and E, zinc (Zn), and selenium (Se) intake. DTAC was estimated using the ferric-reducing ability of plasma assay. Logistic regression models were used to evaluate the associations of DAQS and DTAC with T2DM risk. Restricted cubic splines were used to assess potential non-linear relationships between DTAC and T2DM.

**Results:**

T2DM was observed in 1,238 (9.9%) participants. After adjusting for confounders, compared to the lowest tertiles (T1) of DAQS, the odds ratios (ORs) for T2DM were 1.03 (95% CI 0.82–1.30) in T2 and 0.85 (95% CI 0.68–1.06) in T3 (*P* = 0.010). Compared to T1, the ORs for T2DM in the highest T3 were 0.78 (95% CI 0.67–0.91, *P-trend* = 0.008) for vitamin A, 1.34 (95% CI 1.15–1.56, *P-trend* < 0.001) for vitamin E, 0.83 (95% CI 0.71–0.97, *P-trend* = 0.007) for Se, and 0.86 (95% CI 0.74–1.01, *P-trend* = 0.033) for Zn. Compared to the lowest quartile(Q1) of DTAC, the OR in the highest Q4 was 0.96 (95% CI 0.80–1.17, *P-trend* = 0.024) for T2DM. A non-linear relationship was observed between DATC and T2DM.

**Conclusion:**

Higher DAQS and DATC were associated with a lower T2DM risk, suggesting that consuming antioxidant-rich foods may reduce the T2DM risk.

**Supplementary Information:**

The online version contains supplementary material available at 10.1186/s12986-024-00786-z.

## Introduction

Type 2 diabetes mellitus (T2DM) is a prevalent metabolic disorder characterized by elevated blood glucose levels resulting from impaired insulin secretion, action, or both [1]. According to the International Diabetes Federation, the prevalence of diabetes is rising rapidly worldwide, with over 537 million adults having diabetes. In 2021, diabetes and related complications resulted in 6.7 million deaths worldwide; China has the largest diabetes epidemic in the world, with approximately 141 million people with diabetes [2].

The etiology of T2DM is complex, and its precise pathogenesis remains unclear. Recent evidence indicates that oxidative stress plays a causal and consequential role in many chronic diseases, including T2DM [3–6]. This arises from an imbalance between oxygen-free radical production and antioxidant defenses [7]. Imbalances in reactive oxygen species-mediated redox reactions can lead to chronic cardiovascular diseases, T2DM, cancer, and other disorders [8–10]. One study showed that elevated oxidative stress is a key factor in T2DM onset and progression, often coinciding with increased free radical generation or impairment of antioxidant systems [10]. This disrupts the balance between free radical formation and protection within cells, damaging biomolecules, including lipids, proteins, and DNA [9, 11]. In addition to oxidative stress, dietary habits are another modifiable factor that influences the progression of T2DM. One report suggested [12] that diets that regularly incorporate whole grains, fruits, vegetables, nuts, and seeds could prevent one-fifth of deaths globally. These foods contain not only dietary fiber but also antioxidants. Antioxidants neutralize free radicals at the cellular level to maintain homeostasis [13]. Some antioxidants are derived from non-enzymatic sources such as vitamins, minerals, and bioactive compounds, which are crucial in combating oxidation and oxidative stress [14–16]. Therefore, dietary antioxidant intake may protect against oxidative stress [17]. However, no single antioxidant reflects the total antioxidant capacity of a diet. Hence, indices to determine overall dietary antioxidant capacity are highly valuable [18]. Based on this, dietary total antioxidant capacity (DTAC) has been utilized as a useful tool to quantify dietary antioxidant content [19]. One study that investigated the association between DTAC and plasma antioxidant levels showed a strong correlation between DTAC and plasma antioxidant status [20]. Estimating total antioxidant capacity from an entire diet involves linking the total antioxidant capacity value for each food item with the amount consumed, considering the synergistic effects of dietary antioxidants rather than the effect of single antioxidants. Additionally, dietary antioxidant quality scores (DAQS) [21] are indicators used to assess dietary antioxidant status based on the daily intake of major proven antioxidant nutrients (vitamins A, C, and E, zinc [Zn], and selenium [Se]). The calculation assigns a value of 0 or 1 by comparing the intake and recommended nutrient intake (RNI) and summing the values. DAQS are sensitive and accurate [22]. Further research on indices, such as DTAC and DAQS, can provide greater insights into assessing overall dietary antioxidant capacity and guiding nutritional strategies for managing oxidative stress linked to chronic diseases.

Most studies on dietary antioxidants have focused on exploring the relationship between DAQS and various health outcomes, including bone density [22], cancer [23], cardiorespiratory fitness [24], blood pressure [25], and metabolic syndrome (MetS) [26]. Studies of disease associations with DTAC have mainly focused on hypertension [27], cardiovascular disease [13], MetS [28], and cancer [29].The findings on the relationship between DTAC and health outcomes in diabetic patients have been varied. Some studies have found that adherence to a high DTAC diet improved diabetes complications and atherosclerosis [30], reduced risks of conditions like non-alcoholic fatty liver disease [31] and gestational diabetes [32], while some case-control studies found no associations with outcomes like chronic kidney disease [33]. In China, findings on the association between dietary antioxidants and T2DM remain limited. Human nutrition science has shifted from emphasizing specific nutrients to focusing on overall dietary quality. Given the substantial differences in dietary habits and eating patterns and the relatively monotonous diet of rural populations, it is particularly important to study the relationship between overall dietary antioxidant intake and T2DM risk in the Chinese population.

Therefore, this study aimed to evaluate the associations between DAQS, DTAC, and T2DM in participants in rural Northwest Chinese populations with relatively monotonous diets.

## Materials and methods

### Study population

The data utilized in this cross-sectional study were obtained from the Natural Population Cohort of Northwest China: the Ningxia Project, an ongoing population-based prospective cohort study. The study design has been previously described [34]. Briefly, 15,802 participants from Wuzhong City and Shizuishan City in the Ningxia Hui Autonomous Region of China participated in baseline surveys between March 2018 and May 2019. Demographic information, semi-quantitative food frequency questionnaire (SQFFQ), and blood biochemical measurements were collected from all participants. We excluded 1 936 participants due to incomplete dietary data, and 548 with missing fasting blood glucose(FBG), 848 with missing covariate data, and 3 who reported energy intake levels < 500 kcal or > 5000 kcal. Finally, our analytical sample comprised 12,467 participants. This study was approved by the Ethics Committee of Ningxia Medical University (No. 2018-012), and all participants provided written informed consent.

### General information and anthropometric measurement

General demographic information (sex and age), behavioral lifestyle factors (including smoking, alcohol consumption, and physical activity), medical history, and medication use were collected from the participants through face-to-face interviews by questionnaires. Bioelectrical impedance analysis (InBody 370 system; Biospace, Seoul, Korea) was conducted to measure waist circumference (WC) and body mass index (BMI). Systolic blood pressure (SBP) and diastolic blood pressure (DBP) were measured using OMRON electronic monitors (model HEM-7124; OMRON, Tokyo, Japan). Participants sat for 5 min before obtaining two measurements at 3 to 5 min intervals. The average of the two values was used for analysis.

### Biochemical measurements

Blood samples were collected from the participants in the morning after fasting for over 8 h. The samples were processed within 2 h of collection to separate serum. FBG, total cholesterol (TC), triglycerides (TG), high-density lipoprotein-cholesterol (HDL-C), and low-density lipoprotein-cholesterol (LDL-C) were analyzed on the same day using an automatic biochemical analyzer (BS-430; Mindray, Shenzhen, China).

### Dietary surveys and DAQS and DTAC assessments

Dietary intake was assessed using a validated 69-item SQFFQ [35]. Participants reported their average consumption frequency over the past year for each food item with frequency choices of never or seldom, 1–3 times per month, 1–3 times per week, 4–6 times per week, or day. The frequency choices were assigned the following weights: 0, 0.07, 0.29, 0.71, or 1, respectively. The energy and nutrient content per 100 gram of each food item was determined using nutrition analysis software (Nutrition and Food Safety Institute, Chinese Center for Disease Control and Prevention, version 2.7.5.(k)). Daily energy and nutrient intake was calculated by multiplying the frequency weight by the reported amount consumed, which was then multiplied by the energy/nutrient density per gram for each food item. The nutrient intake was adjusted using the residual method.

DAQS were calculated based on the intake of five key antioxidant nutrients [21]: vitamins A, C, and E, Zn, and Se. The intake of these nutrients was compared with the RNI or adequate intake (AI) for the Chinese population, published by the China Nutrition Society in June 2013. Each nutrient was evaluated individually and assigned a score of 0 or 1. A score of 0 was given if the intake was less than two-thirds of the RNI (or AI). A score of 1 was assigned if the intake was greater than or equal to two-thirds of the RNI (or AI). The scores for the five nutrients were summed to obtain the total DAQS, ranging from 0 (poor quality) to 5 (high quality). The daily intake of antioxidant nutrients in the study population is shown in Table S1 (Additional file 1: Table S1).

To calculate the DTAC, the daily intake of each food item was multiplied by its antioxidant potential, and the results were summed to obtain the DTAC for each participant. The antioxidant potential of foods was determined using the ferric-reducing ability of plasma assay, as published in the Antioxidant Food Table by Carlsen et al., which contains the antioxidant contents of over 3100 types of foods and beverages [19]. The antioxidant value of the nearest comparable food was assigned for any food item without available antioxidant data. The total DTAC for each participant was calculated by multiplying the daily intake of each food by its antioxidant potential value and summing all foods consumed. The contributions of the 18 matched food items to the antioxidant indicators are shown in Table S2 (Additional file 1: Table S2).

### Definition of variables

T2DM was defined based on the following criteria according to the Guidelines for the Prevention and Treatment of T2DM in China (2020 edition): FBG ≥ 7.0 mmol/L; self-reported previous diagnosis of T2DM; or current use of glucose-lowering medication. Physical activity was categorized as low, moderate, or high and was assessed using the International Physical Activity Questionnaire [36]. For smoking and alcohol consumption, non-users were defined as having had no use in the past year, while any other frequency of use was defined as yes. Health supplement intake was defined as the consumption of at least one of the following: fish oil/cod liver oil, vitamins, calcium/iron/Zn, ginseng products, or other supplements.

### Statistical analysis

Participants were categorized into tertiles (T1-T3) based on DAQS and into quartiles(Q1-Q4) based on DTAC. Continuous variables, including age, WC, BMI, SBP, DBP, FBG, TC, TG, HDL-C, and LDL-C, are presented as mean ± standard deviation (SD) for normally distributed variables and compared between groups using Analysis of Variance (ANOVA). Categorical variables, including sex, smoking, alcohol consumption, education level, physical activity, disease of hypertension, and T2DM, are presented as numbers (%) and compared between groups using Chi-square tests. Associations between DAQS, DTAC, and T2DM were evaluated using logistic regression models after adjusting for confounders. The results are expressed as odds ratios (ORs) and 95% confidence intervals (CIs). *P* for trends were tested using the median of each category as a continuous variable. Furthermore, a restricted cubic spline was used to evaluate the nonlinear association between DTAC and T2DM by setting DTAC-restricted nodes to the 5th, 35th, 65th, and 95th percentiles in R software version 4.2.3. Other statistical analyses were performed using SPSS software version 23.0 (SPSS Inc., Chicago, IL, USA) and *P* < 0.05 was considered statistically significant.

## Results

Of the 12,467 participants, 1 238 had T2DM, with a prevalence of 9.9%. As shown in Table 1, with increasing DAQS tertiles, there was a decreasing trend in age, WC, SBP, DBP and T2DM prevalence (*P* < 0.01). Similarly, with increasing DTAC levels, there was a decreasing trend in TC levels (*P* < 0.01) (Table 2). In addition, the differences in sex, education level, physical activity, smoking, alcohol consumption, TG, LDL-C, and HDL-C levels were statistically significant across DAQS and DTAC groups(*P* < 0.01) (Tables 1 and 2).

**Table 1:** General characteristics study participants according to tertiles of DAQS

Characteristics	Total (*n* = 12,467)	T1 (0–1) (*n* = 1173)	T2 (2–3) (*n* = 3963)	T3 (4–5) (*n* = 7331)	*P*-value
Age (years)	56.86 ± 9.93	59.62 ± 9.28	56.76 ± 9.74	56.48 ± 10.07	< 0.001
Sex					< 0.001
Male	4961(39.8)	1132(96.5)	1175(29.6)	2654(36.2)	
Female	7 506(60.2)	41(3.5)	2 788(70.4)	4 677(63.8)	
Smoking					0.002
Yes	1892(15.2)	448(38.2)	533(13.4)	911(12.4)	
No	10,575(84.8)	725(61.8)	3430(86.6)	6420(87.6)	
Alcohol consumption					< 0.001
Yes	2973(23.8)	603(51.4)	1159(29.2)	1211(16.5)	
No	9494(76.2)	570(48.6)	2804(70.8)	6120(83.5)	
Educational level					< 0.001
Primary school or below	8512(68.3)	730(62.2)	2683(67.7)	5099(69.6)	
Middle school	3886(31.2)	436(37.2)	1258(31.7)	2192(29.9)	
college or higher	69(0.5)	7(0.6)	22(0.6)	40(0.5)	
Physical activity					< 0.001
Low	3904(31.3)	245(20.9)	892(22.5)	2767(37.7)	
Moderate	6587(52.8)	804(68.5)	2464(62.2)	3319(45.3)	
High	1976(15.9)	124(10.6)	607(15.3)	1245(17.0)	
WC (cm)	87.09 ± 9.82	88.84 ± 10.66	87.03 ± 9.74	86.85 ± 9.69	< 0.001
BMI (kg/m^2^)	24.95 ± 3.43	25.04 ± 3.37	24.87 ± 3.46	24.97 ± 3.43	0.239
SBP (mmHg)	135.16 ± 19.58	140.38 ± 20.00	139.00 ± 20.90	132.26 ± 18.20	< 0.001
DBP (mmHg)	83.14 ± 12.65	86.49 ± 13.61	84.84 ± 13.12	81.68 ± 12.00	< 0.001
FBG (mmol/L)	5.68 ± 1.94	5.75 ± 1.64	5.77 ± 2.08	5.62 ± 1.90	< 0.001
TC (mmol/L)	4.85 ± 1.25	4.79 ± 0.96	4.91 ± 1.04	4.83 ± 1.38	0.001
TG (mmol/L)	1.71 ± 1.17	1.64 ± 1.21	1.64 ± 1.13	1.76 ± 1.19	< 0.001
HDL-C (mmol/L)	1.35 ± 0.40	1.30 ± 0.35	1.39 ± 0.35	1.34 ± 0.43	< 0.001
LDL-C (mmol/L)	2.85 ± 1.14	2.63 ± 0.75	2.79 ± 1.51	2.93 ± 0.92	< 0.001
Hypertension					< 0.001
Yes	6118(49.1)	723(61.6)	2201(55.5)	3194(43.6)	
No	6349(50.9)	450(38.4)	1762(44.5)	4137(56.4)	
T2DM					< 0.001
Yes	1238(9.9)	131(11.2)	434(11.0)	673(9.2)	
No	11,229(90.1)	1042(88.8)	3529(89.0)	6658(90.8)	

Data are presented as mean ± SD or n (%) as indicated

WC waist circumference, BMI body mass index, SBP systolic blood pressure, DBP diastolic blood pressure, FBG fasting blood glucose, TC total cholesterol, TG triglyceride, HDL-C high-density lipoprotein-cholesterol, LDL-C low-density lipoprotein-cholesterol

**Table 2:** General characteristics study participants according to quartiles(Q) of DTAC

Characteristics	Q1 (< 1.34) (*n* = 3 131)	Q2 (1.34–2.43) (*n* = 3 096)	Q3 (2.43–4.06) (*n* = 3 125)	Q4 (> 4.06) (*n* = 3 115)	*P*-value
Age (years)	57.04 ± 10.13	58.01 ± 9.66	56.37 ± 9.82	56.05 ± 10.00	< 0.001
Sex					< 0.001
Male	1084(34.6)	1309(42.3)	1246(39.9)	1322(42.4)	
Female	2047(65.4)	1787(57.7)	1879(60.1)	1793(57.6)	
Smoking					0.002
Yes	450(14.4)	536(17.3)	452(14.5)	454(14.6)	
No	2681(85.6)	2560(82.7)	2673(85.5)	2661(85.4)	
Alcohol consumption					< 0.001
Yes	1078(34.4)	858(27.7)	525(16.8)	512(16.4)	
No	2053(65.6)	2238(72.3)	2600(83.2)	2603(83.6)	
Educational level					< 0.001
Primary school or below	2137(68.3)	2221(71.7)	2093(67.0)	2061(66.1)	
Middle school	980(31.3)	865(28.0)	1011(32.3)	1030(33.1)	
College or higher	14(0.4)	10(0.3)	21(0.7)	24(0.8)	
Physical activity					< 0.001
Low	1156(36.9)	795(25.7)	757(24.2)	1196(38.4)	
Moderate	1716(54.8)	1907(61.6)	1739(55.7)	1225(39.3)	
High	259(8.3)	394(12.7)	629(20.1)	694(22.3)	
WC (cm)	86.78 ± 9.85	87.05 ± 9.86	87.35 ± 9.80	87.20 ± 9.74	0.126
BMI (kg/m^2^)	25.02 ± 3.44	24.89 ± 3.41	24.96 ± 3.47	24.91 ± 3.41	0.455
SBP (mmHg)	136.04 ± 19.74	137.98 ± 20.14	135.68 ± 19.73	130.97 ± 17.96	< 0.001
DBP (mmHg)	84.33 ± 12.89	83.59 ± 12.76	83.26 ± 12.78	81.38 ± 11.96	< 0.001
FBG (mmol/L)	5.54 ± 1.91	5.81 ± 1.99	5.75 ± 1.69	5.61 ± 2.11	< 0.001
TC (mmol/L)	4.94 ± 0.97	4.88 ± 1.06	4.81 ± 0.99	4.78 ± 1.78	< 0.001
TG (mmol/L)	1.68 ± 1.06	1.68 ± 1.19	1.70 ± 1.15	1.79 ± 1.28	0.001
HDL-C (mmol/L)	1.34 ± 0.32	1.38 ± 0.34	1.37 ± 0.34	1.32 ± 0.54	< 0.001
LDL-C (mmol/L)	2.68 ± 1.64	2.83 ± 0.80	2.95 ± 0.89	2.97 ± 0.98	< 0.001
Hypertension					< 0.001
Yes	1624(51.9)	1669(53.9)	1582(50.6)	1243(39.9)	
No	1507(48.1)	1427(46.1)	1543(49.4)	1872(60.1)	
T2DM					< 0.001
Yes	255(8.1)	384(12.4)	352(11.3)	247(7.9)	
No	2876(91.9)	2712(87.6)	2773(88.7)	2868(92.1)	

Data are presented as mean ± SD or n (%) as indicated

WC waist circumference, BMI body mass index, SBP systolic blood pressure, DBP diastolic blood pressure, FBG fasting blood glucose, TC total cholesterol, TG triglyceride, HDL-C high-density lipoprotein-cholesterol, LDL-C low-density lipoprotein-cholesterol

Tables 3 and 4 present the logistic regression analysis to explore the associations between DAQS, DTAC, and T2DM. As shown in Table 3, for DAQS, compared to the lowest T1, the unadjusted OR for T2DM in the highest T3 was 0.80 (95% CI 0.66–0.98, *P* = 0.002). After adjusting for potential confounding factors, the ORs for T2DM in T3 remained statistically significant, with values 0.84 (95% CI 0.68–1.05, *P* = 0.007) in Model 2 and 0.85 (95% CI 0.68–1.06, *P* = 0.010) in Model 3. Additionally, compared to the lowest T1, the adjusted ORs for T2DM in T3 were 0.78 (95% CI 0.67–0.91, *P-trend* = 0.008) for vitamins A, 0.83 (95% CI 0.71–0.97, *P-trend* = 0.007) for Se, and 0.86 (95% CI 0.74–1.01, *P-trend* = 0.033) for Zn. However, with increasing levels of vitamin E, the adjusted ORs for T2DM increased to 1.19 (95% CI 1.02–1.39) in T2 and 1.34 (95% CI 1.15–1.56) in T3(*P-trend* < 0.001).

**Table 3:** Logistic regression analysis models investigating the associations between DAQS and T2DM

Characteristics	T1	T2	T3	*P*-value^a^
DAQS	0–1	2–3	4–5	
Model 1	1.00 (ref)	0.98(0.80–1.20)	0.80(0.66–0.98)	0.002
Model 2		1.02(0.81–1.28)	0.84(0.68–1.05)	0.007
Model 3		1.03(0.82–1.30)	0.85(0.68–1.06)	0.010
Vitamin A	< 233.55	233.55-4762.65	> 4762.65	
Model 1	1.00 (ref)	0.82(0.71–0.94)	0.79(0.68–0.91)	0.012
Model 2		0.81(0.70–0.94)	0.80(0.69–0.93)	0.038
Model 3		0.85(0.74–0.99)	0.78(0.67–0.91)	0.008
Vitamin C	< 35.87	35.87–59.22	> 59.22	
Model 1	1.00 (ref)	1.03(0.89–1.19)	1.09(0.95–1.26)	0.217
Model 2		1.05(0.91–1.21)	1.18(1.02–1.37)	0.022
Model 3		1.05(0.90–1.21)	1.14(0.98–1.33)	0.073
Vitamin E	< 92.49	92.49-161.61	> 161.61	
Model 1	1.00 (ref)	1.20(1.03–1.40)	1.49(1.29–1.73)	< 0.001
Model 2		1.16(1.00-1.35)	1.31(1.13–1.52)	< 0.001
Model 3		1.19(1.02–1.39)	1.34(1.15–1.56)	< 0.001
Se	< 43.24	43.24–73.91	> 73.91	
Model 1	1.00 (ref)	0.98(0.85–1.13)	0.81(0.70–0.94)	0.003
Model 2		1.00(0.87–1.15)	0.85(0.73–0.99)	0.020
Model 3		1.03(0.89–1.19)	0.83(0.71–0.97)	0.007
Zn	< 8.45	8.45–11.59	> 11.59	
Model 1	1.00 (ref)	1.01(0.88–1.16)	0.83(0.72–0.96)	0.006
Model 2		1.03(0.89–1.18)	0.88(0.76–1.02)	0.062
Model 3		1.05(0.91–1.22)	0.86(0.74–1.01)	0.033

DAQS dietary antioxidant quality score, Se selenium, Zn zinc

Model 1: unadjusted

Model 2: adjusted for age, sex, smoking status, alcohol consumption, and physical activity

Model 3: model 2 with additional adjustment for BMI, WC, TG, HDL-C, hypertension and health supplement intake

a *P*-value:only DAQS adopted *P*-value, while the others all adopted *P*-trend

**Fig. 1:**
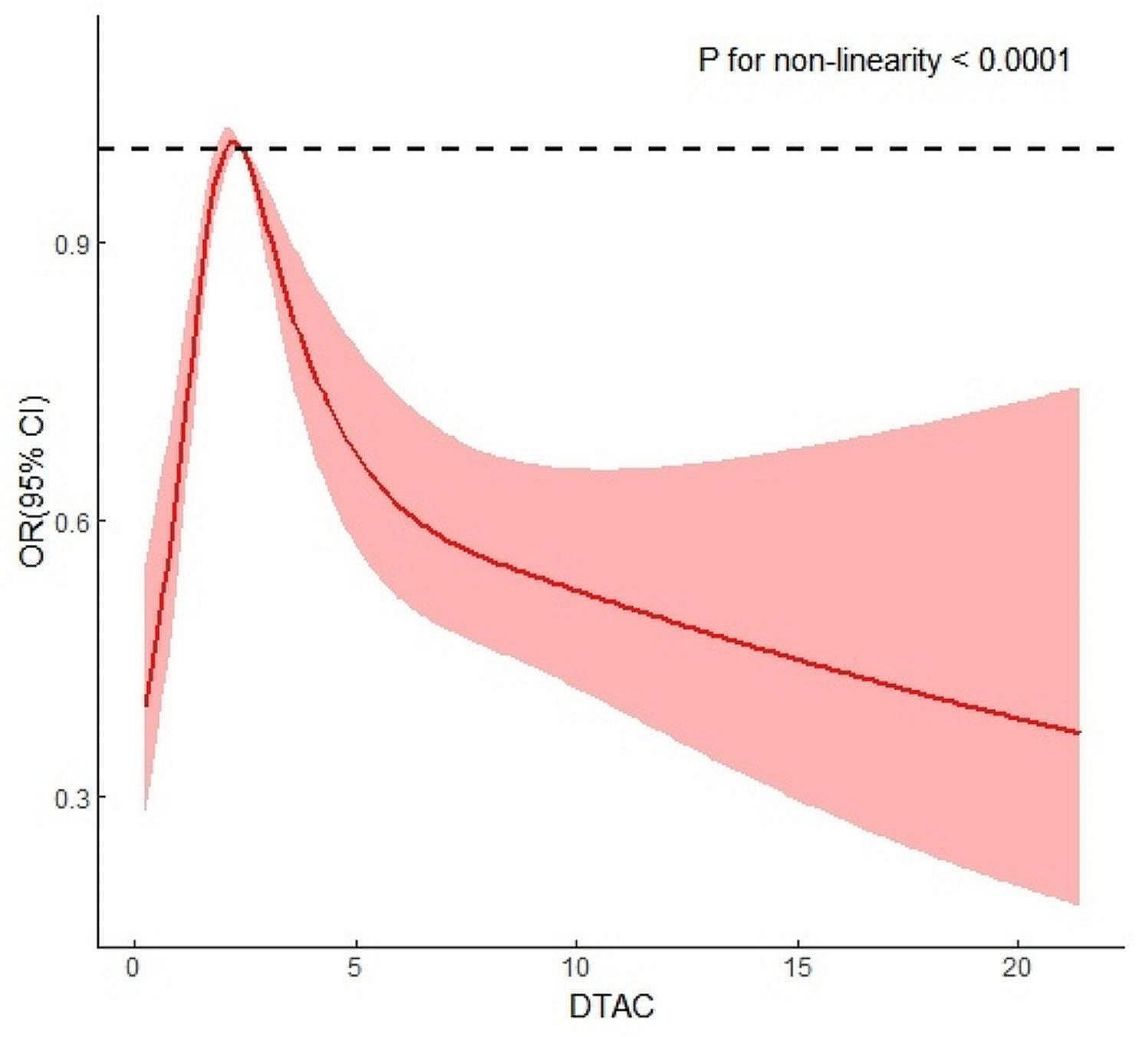
Restricted cubic spline of the relationship of T2DM with DTAC The dash line represents the OR equal to 1. The ORs was adjusted for age, sex, smoking status, alcohol consumption, physical activity,BMI, WC, TG, HDL-C hypertension and health supplement intake

Compared to the lowest Q4 in Table 4, as DTAC levels increased, the ORs for T2DM were 1.51 (95% CI 1.27–1.79), 1.39 (95% CI 1.16–1.66) and 0.96 (95% CI 0.80–1.17), respectively, (*P-trend* = 0.024). Furthermore, the restricted cubic spline showed a nonlinear relationship between DTAC and T2DM (Fig. 1).

**Table 4:** Logistic regression analysis models investigating the associations between DTAC and T2DM

Characteristics	Q1	Q2	Q3	Q4	*P*-trend
DATC	< 1.34	1.34–2.43	2.43–4.06	> 4.06	
Model 1	1.00 (ref)	1.60(1.35–1.89)	1.43(1.21–1.70)	0.97(0.81–1.17)	0.018
Model 2		1.53(1.29–1.81)	1.43(1.20–1.70)	0.99(0.82–1.19)	0.036
Model 3		1.51(1.27–1.79)	1.39(1.16–1.66)	0.96(0.80–1.17)	0.024

DTAC dietary total antioxidant capacity

Model 1: unadjusted

Model 2: adjusted for age, sex, smoking status, alcohol consumption, and physical activity

Model 3: model 2 with additional adjustment for BMI, WC, TG, HDL-C, hypertension and health supplement intake

## Discussion

This cross-sectional study found that higher intakes of DAQS and DTAC were associated with a lower risk of T2DM. Intake of certain individual nutrients, including vitamin A, Se, and Zn, can also be associated with reduced T2DM risk.

Recent research has emphasized the effect of specific antioxidant nutrients on health. However, information on the cumulative and interactive effects of dietary antioxidants is limited. Similarly, minimal research has been conducted on the relationship between DAQS and T2DM risk. Further studies on the association between total antioxidant vitamin and mineral intakes and T2DM are warranted. One prospective cohort study [37] that investigated DAQS and all-cause and cause-specific mortality among adults with T2DM found higher DAQS significantly correlated with reduced all-cause mortality (HR=0.70, 95% CI 0.53–0.92 for the highest vs. the lowest DAQS quartiles). This indicates that adequate intake of antioxidant micronutrients may reduce mortality in patients with T2DM. However, in a study that involved an obese Iranian population, [25] higher DAQS were only positively associated with a lower DBP, with no significant relationships observed between DAQS and serum lipids, glycemic markers, or insulin resistance biomarkers. A randomized, double-blind, placebo-controlled primary prevention trial undertaken in France examined the effects of 7.5 years of antioxidant supplementation on the incidence of MetS [38]. The correlations between baseline serum antioxidant levels and prospective MetS risk were also assessed. MetS incidence was negatively associated with baseline serum-carotene and vitamin C levels but positively associated with Zn levels. Although not statistically significant, increased baseline vitamin E and Se levels were associated with an increased risk of MetS. Another analysis of European populations found a strong negative association between plasma vitamin C levels and T2DM risk [39]. Compared with the bottom quintile of plasma vitamin C, the top quintile had 62% lower odds of developing T2DM. However, a study that involved Lebanese adults found that plasma Se levels were positively correlated with all MetS components, including glucose (*r* = 0.105, *P* = 0.037) [40]. In contrast, plasma Zn levels did not correlate with any of these components. Therefore, findings from research on various nutrients and T2DM risk are inconsistent. Here, we found vitamin A, Se, Zn (only in Models 1 and 3) and DAQS to be negatively correlated with T2DM risk, while vitamins C (only in Model 2) and E were positively correlated. Compared with the Chinese RNI, the highest tertile had higher vitamin A and Se levels. Antioxidants are primarily derived from animal organs, seafood, and meat. However, this study group had high vitamin A intake due to the consumption of animal organs and red meat, and some studies have suggested uncertainty about how T2DM affects vitamin A metabolism [41]. Dietary vitamin A intake in patients with T2DM may vary depending on factors such as the study population, dietary habits, food diversity, regional socioeconomic status, and genetics. Vitamin E is a fat-soluble vitamin primarily obtained from vegetable oils, nuts, and seeds. In our study population, the vitamin E levels in the lowest tertile group were significantly higher than the AI of 14 mg for Chinese residents because of the large amounts of oil and salt used in cooking. Regardless of sex, 0% of the participants had vitamin E levels below two-thirds of the AI. While one study showed that vitamin E intake reduced the T2DM risk [42], another study showed that high doses may promote oxidative stress [43]. This may explain the positive correlation between vitamin E levels and T2DM risk observed in our study. Vitamin C is a water-soluble vitamin primarily obtained from fruits and vegetables. Our study participants had a low intake of these food sources, with male and female participants having intakes below two-thirds of the RNI at 73.8% and 71.2%, respectively. Research suggests that FFQs may overestimate fruit and vegetable consumption compared with more detailed dietary assessments [44]. Therefore, the actual intake of fruits and vegetables, and thus vitamin C, in our participants was likely to be even lower than that suggested by the results, indicating that more individuals had inadequate vitamin C intake below two-thirds of the RNI. A study [45] have indicated that vitamin C intake and serum vitamin C levels were negatively correlated with fasting blood glucose. Low vitamin C intake and/or insufficient serum levels can increase mortality risk in patients with T2DM (HR 1.25, 95% CI 1.05–1.49 and 1.84, 95% CI 1.10–3.08). The study recommends vitamin C intake, including supplementing 500–1000 mg/day of vitamin C, as potentially more beneficial for American adults with diabetes or prediabetes. Based on this study, we speculate that this may be why the vitamin C results were not significant. If adequate vitamin C intake is consumed, it may be inversely associated with the risk of developing T2DM. Currently, research findings on Zn are inconsistent. A randomized trial [37] found that Zn was positively associated with the risk of MetS. However, prospective evidence showed [46] an inverse association between Zn intake and T2DM risk. A clinical controlled trial found [47] that Zn supplementation reduced blood glucose and insulin resistance while improving β-cell function. Another clinical controlled trial [48] in obese women came to the same conclusion. In our study, we also found that Zn was inversely associated with the risk of T2DM (only significant in Model 1 and Model 3).

The epidemiological evidence on the relationship between DTAC and T2DM risk is limited. However, some large cohort studies have reported such correlations. One prospective study in French women found that high DTAC levels were associated with lower T2DM risk (HR=0.73, highest quintile) [49]. Another cohort study that involved adults aged ≥ 45 years also found that a higher DTAC correlated with a lower T2DM risk [50]. That study observed a correlation between DTAC and prediabetes in men but not in women, whereas the association with insulin resistance was slightly stronger in women. This potential sex difference may be related to differences in visceral fat. A Hertfordshire cohort study in the British population found negative correlations between DTAC and fasting insulin, insulin resistance, and glucose tolerance using four DTAC measurement methods [51]. However, all the aforementioned studies were conducted involving non-Chinese populations, and currently, evidence regarding the association between DTAC use and T2DM risk in Chinese populations is lacking. In this study, although the risk of T2DM gradually decreased with increasing DTAC levels, DTAC was a risk factor for T2DM in the Q2 and Q3 groups and only a protective factor in the highest quartile Q4 group. There are two potential reasons for this finding. First, the dietary patterns in populations assessed in other studies differ greatly from those in our study region. In those previous study populations, more antioxidant-rich foods, such as coffee, nuts, red wine, deep-sea fish, and olive oil, were consumed; however, our study population rarely consumed these types of antioxidants. A study conducted among the Polish population found a significant inverse relationship between DTAC and prediabetes prevalence, with the Q1 group having < 8.37 mmol/day and the Q4 group having > 14.51 mmol/day [52]; whereas our study had significantly lower DTAC values, with only < 1.34 mmol/day in the Q1 group and > 4.06 mmol/day in the Q4 group. Second, while databases used to assess the total antioxidant content of the diet have been established in many other countries, some foods lack accurate antioxidant potential values, with similar foods being used as substitutes. Accordingly, further research is warranted to investigate the effects of DAQS and DTAC on T2DM in different populations.

Our study provides epidemiological evidence concerning the relationship between antioxidants, DAQS, DTAC, and T2DM risk in the dietary intake of the Chinese population. However, our study has some limitations. First, because our study participants were from rural areas in Northwest China, their daily food consumption was relatively uniform and single, which may limit the generalizability of the results. Second, we used an SQFFQ to estimate the participants’ dietary intake frequency over the past year, which may introduce subjective recall bias and pose challenges in accurately assessing dietary habits. Third, although we extensively adjusted for all potential confounding variables, we cannot completely exclude unmeasured confounding factors. Finally, as with all cross-sectional studies, we could not establish a causal relationship between the DAQS, DTAC, and T2DM.

In this study of rural populations in northwest China, significant inverse associations were observed between DAQS, DTAC and decreased risk of T2DM. A non-linear relationship was found between DATC and T2DM. These findings suggest that incorporating antioxidant-rich foods into the diet may help reduce the risk of T2DM.

### Electronic supplementary material

Below is the link to the electronic supplementary material.

Supplementary Material: Table S1. Daily intake of the antioxidant nutrients in the study population; Table S2. Food contributors to the dietary total antioxidant capacity.

## Data Availability

No datasets were generated or analysed during the current study.

## Abbreviations

T2DM: Type 2 diabetes mellitus
DAQS: Dietary antioxidant quality scores
DTAC: Dietary total antioxidant capacity
Zn: Zinc
Se: Selenium
RNI: Recommended nutrient intake
MetS: Metabolic syndrome
SQFFQ: Semi-quantitative food frequency questionnaire
FBG: Fasting blood glucose
WC: Waist circumference
BMI: Body mass index
SBP: Systolic blood pressure
DBP: Diastolic blood pressure
TC: Total cholesterol
TG: Triglycerides
HDL-C: High-density lipoprotein-cholesterol
LDL-C: Low-density lipoprotein-cholesterol
AI: Adequate intake
ORs: Odds ratios
CIs: Confidence intervals
