# Supplementary material for: Association between dietary antioxidant capacity and type 2 diabetes mellitus in Chinese adults: a population-based cross-sectional study
Source: Nutr Metab (Lond). 2024 Mar 29;21:16. doi: 10.1186/s12986-024-00786-z (PMC10981302; doi:10.1186/s12986-024-00786-z)
Supplement: Supplementary file 1 — Supplementary Material: Table S1. Daily intake of the antioxidant nutrients in the study population; Table S2. Food contributors to the dietary total antioxidant capacity. [file 12986_2024_786_MOESM1_ESM.docx]

**Table S1. Daily intake of the antioxidant nutrients in the study population**

| Characteristics | Males | Females |
| --- | --- | --- |
|  | % sample under 2/3 RNI (AI) | % sample under 2/3 RNI (AI) |
| Vitamin A (μg RAE/day) | 45.4 | 41.1 |
| Vitamin C (mg/day) | 73.8 | 71.2 |
| Vitamin E (mg/day) | 0 | 0 |
| Zn (mg/day) | 34.6 | 0.6 |
| Se (μg/day) | 28.4 | 24.9 |

Zn zinc, Se selenium.

**Table S2. Food contributors to the dietary total antioxidant capacity**

| Food Contribution | % Contribution to DTAC | | |
| --- | --- | --- | --- |
|  | Males | Females | Total |
| Tea | 1.45 | 0.94 | 1.14 |
| Alcohol | 1.41 | 0.38 | 0.79 |
| Staple food | 7.95 | 8.50 | 8.28 |
| Meat and meat products | 20.16 | 22.26 | 21.42 |
| Fish/Seafood | 0.39 | 0.37 | 0.37 |
| Eggs and egg products | 0.14 | 0.10 | 0.12 |
| Fresh vegetables | 29.30 | 28.90 | 29.06 |
| Fresh fruits | 6.62 | 6.65 | 6.64 |
| Potatoes and sweet potatoes | 4.69 | 4.72 | 4.71 |
| Legumes and soy products | 3.14 | 3.27 | 3.22 |
| Dairy products | 0.55 | 0.62 | 0.59 |
| Dried vegetables | 1.02 | 0.94 | 0.97 |
| Dried fruits | 0.28 | 0.23 | 0.25 |
| Nuts and seeds | 7.83 | 8.11 | 8.00 |
| Goji Berries | 0.16 | 0.14 | 0.15 |
| Spices | 7.30 | 6.64 | 6.90 |
| Oils | 7.06 | 6.78 | 6.90 |
| Others | 0.55 | 0.45 | 0.49 |
